# Supplementary figures and images for: Structure–Functional Examination of Novel Ribonucleoside Hydrolase C (RihC) from Limosilactobacillus reuteri LR1
Source: Int J Mol Sci. 2023 Dec 30;25(1):538. doi: 10.3390/ijms25010538 (PMC10778931; doi:10.3390/ijms25010538)

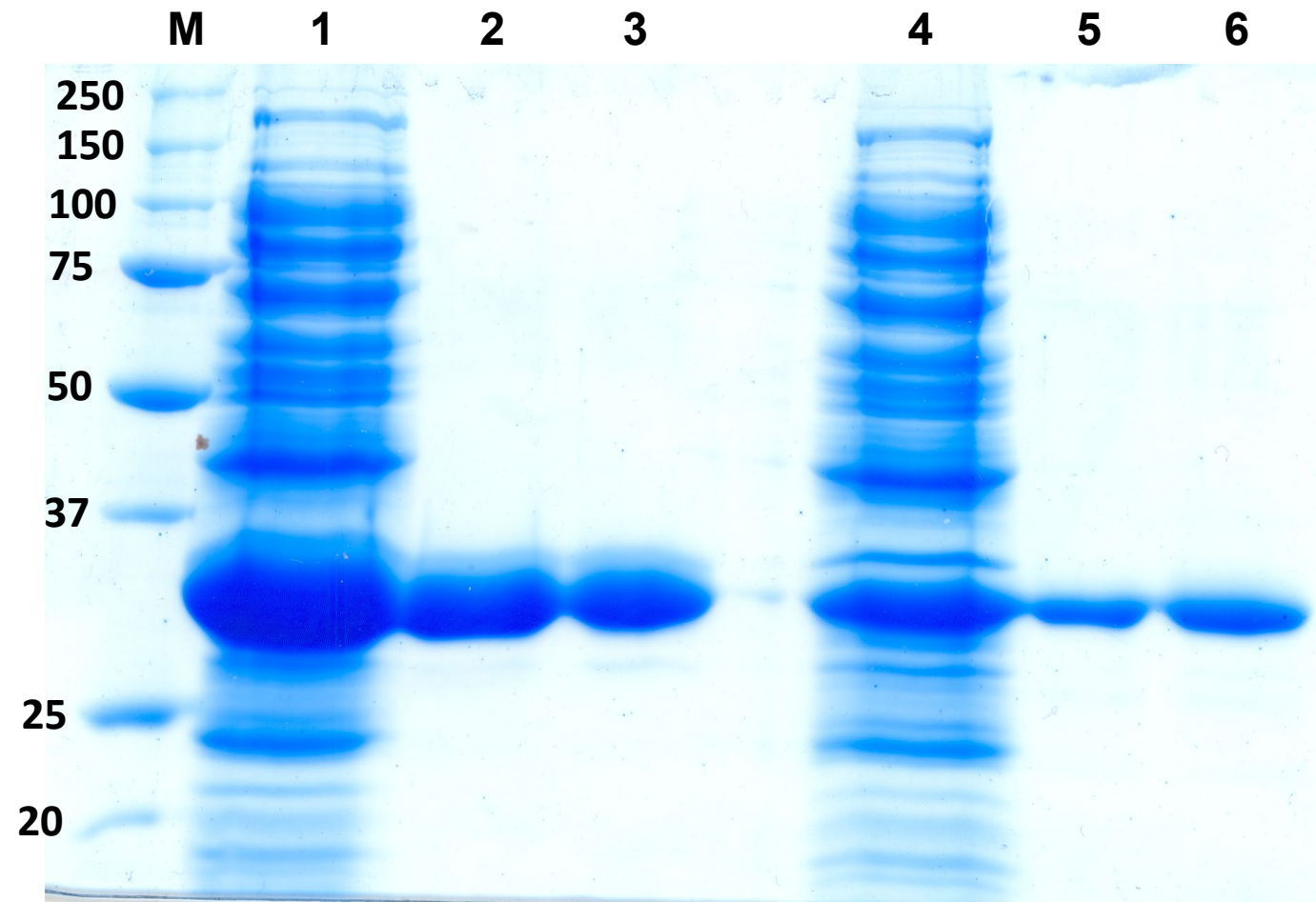

Supplement: Supplementary file 1 [file ijms-25-00538-s001.zip › Figure S1.pdf]

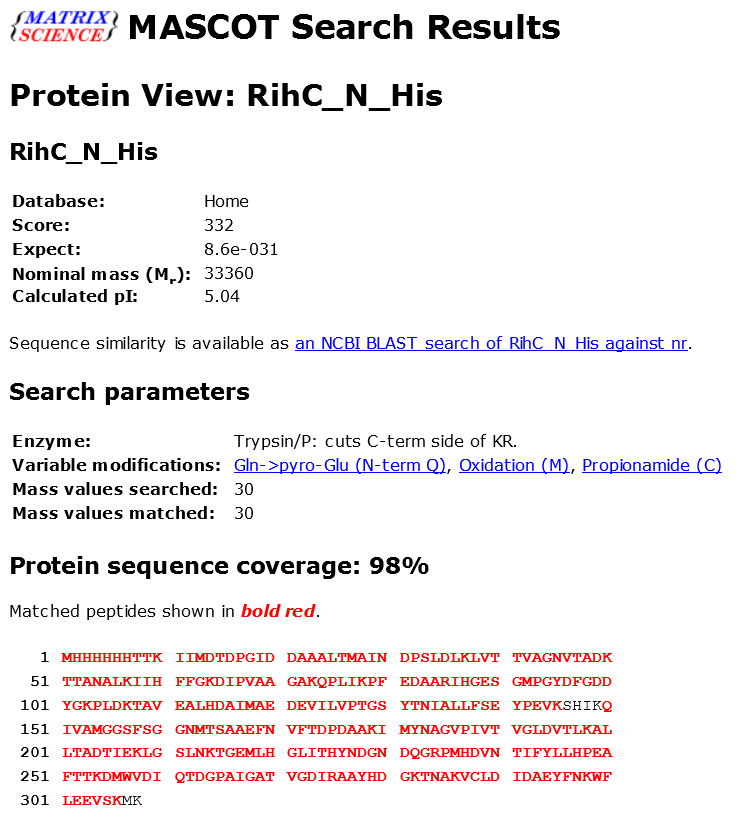

Supplement: Supplementary file 1 [file ijms-25-00538-s001.zip › Figure S2.PNG]

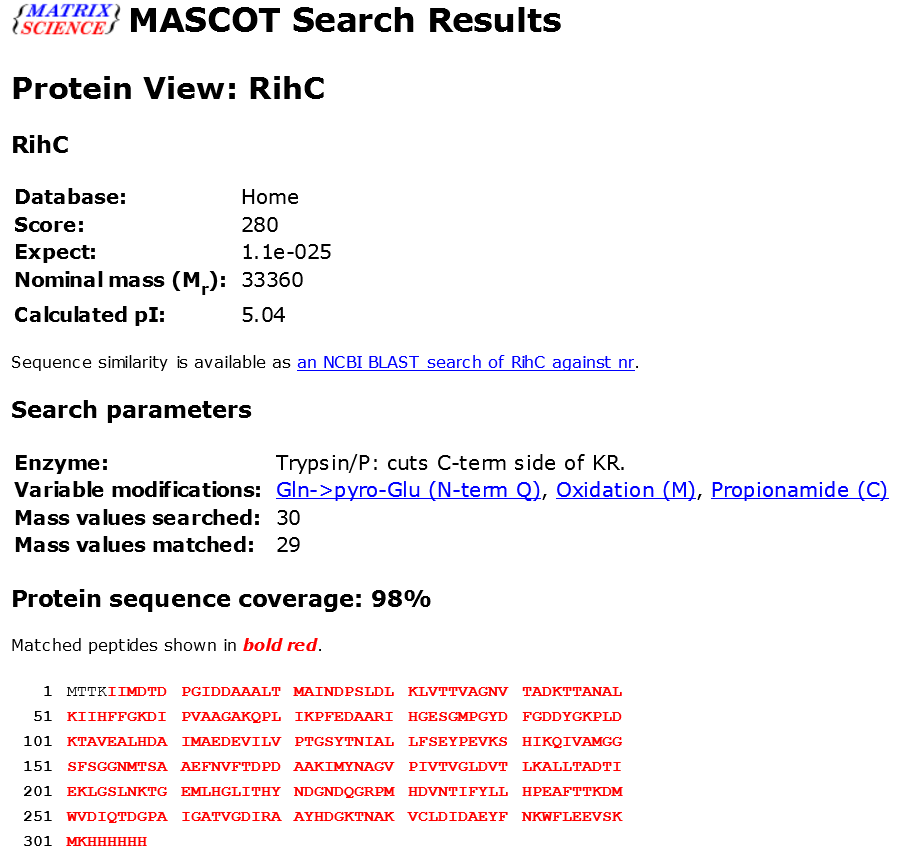

Supplement: Supplementary file 1 [file ijms-25-00538-s001.zip › Figure S3.PNG]

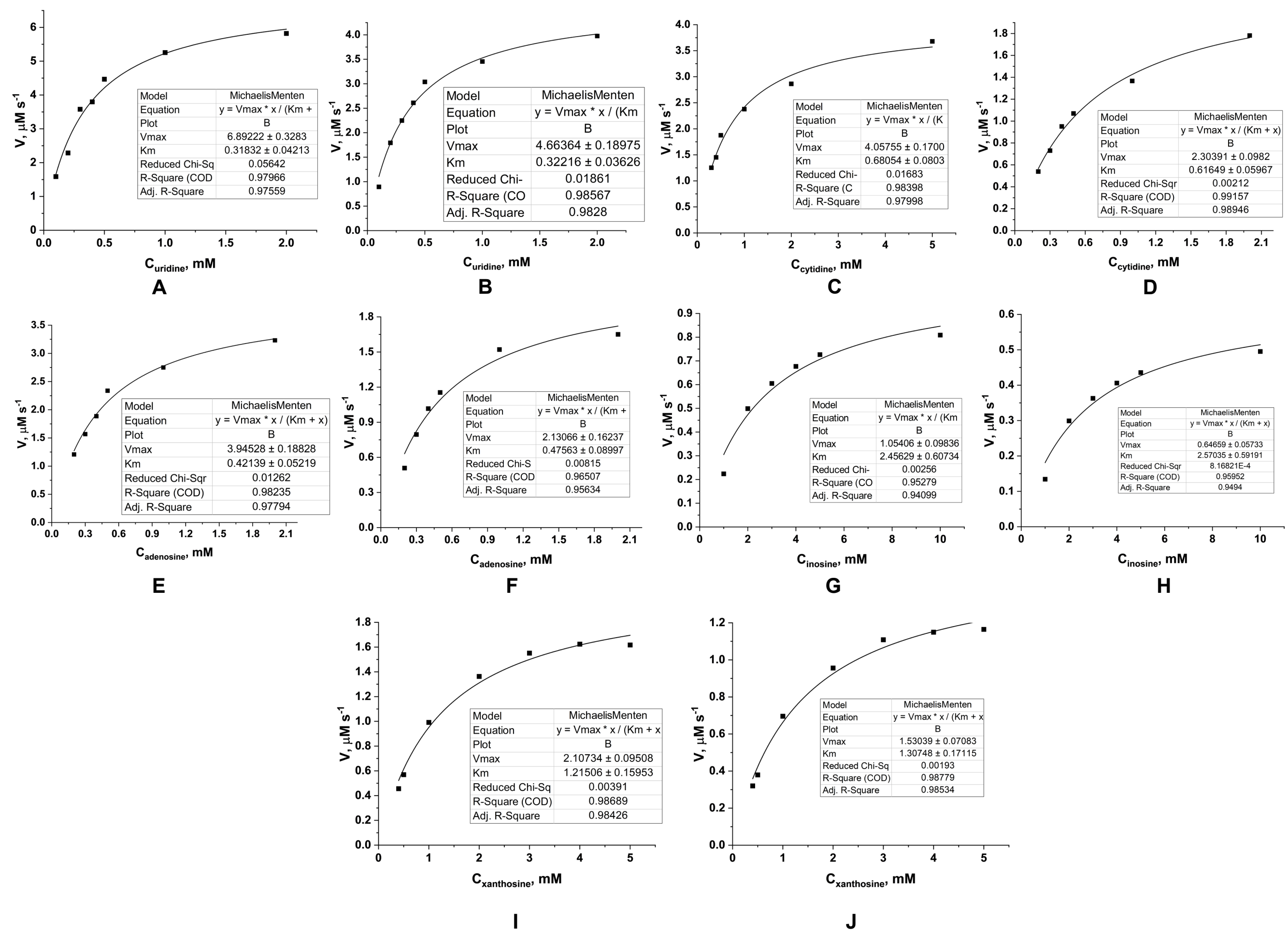

Supplement: Supplementary file 1 [file ijms-25-00538-s001.zip › FIgure S4.pdf]

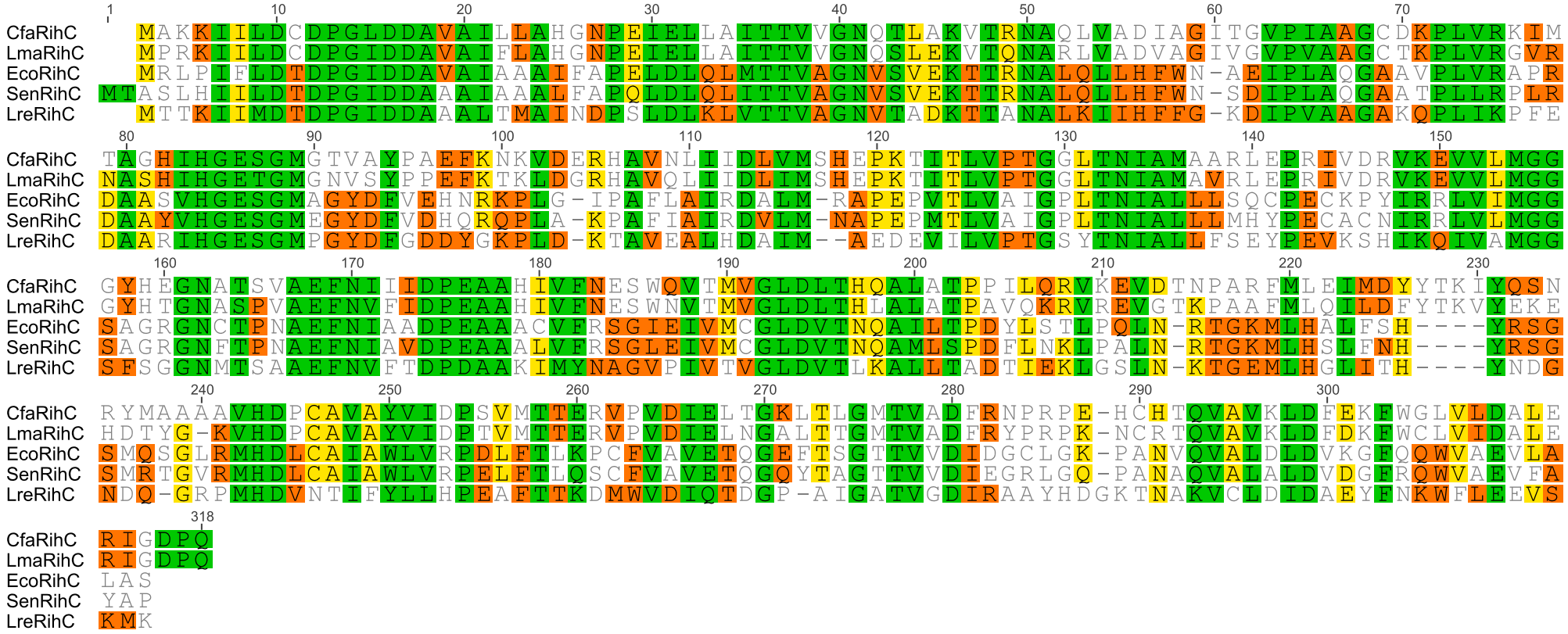

Supplement: Supplementary file 1 [file ijms-25-00538-s001.zip › Figure S5.pdf]

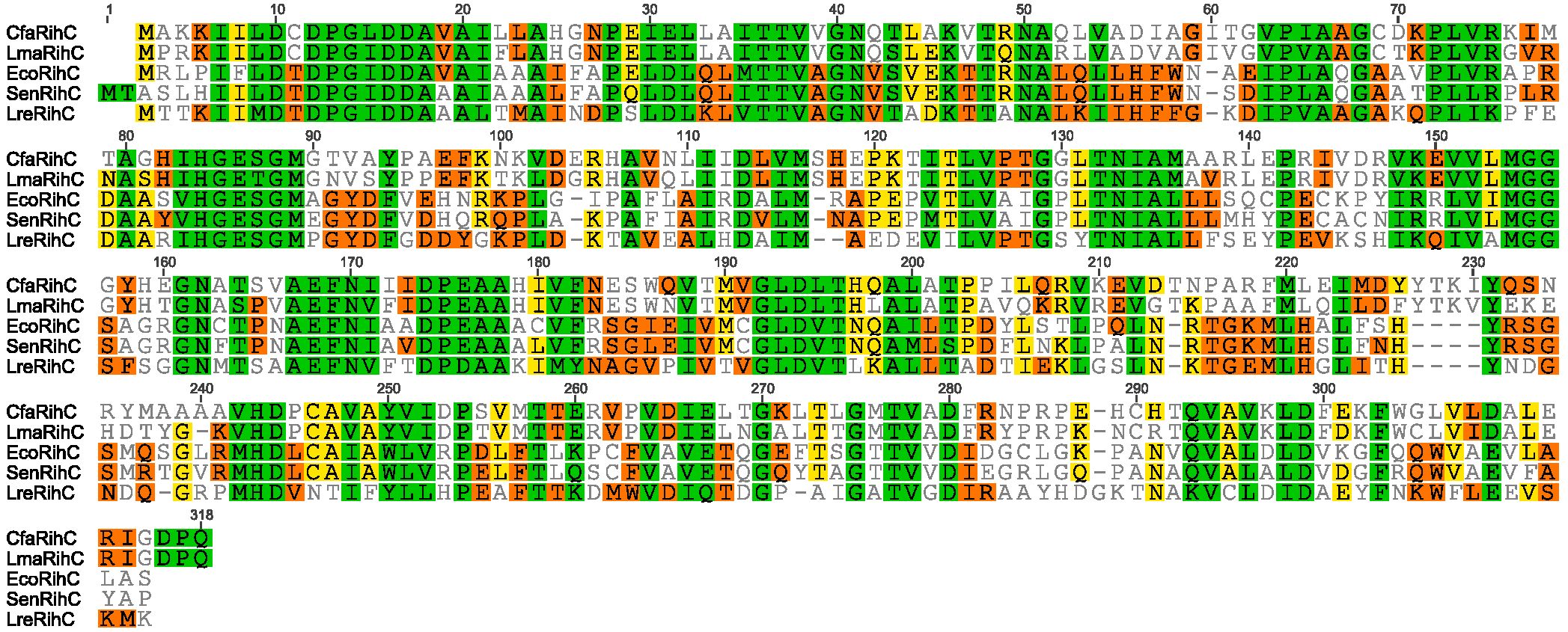

Supplement: Supplementary file 1 [file ijms-25-00538-s001.zip › Figure S5.tif]
